# Message in a Microbottle: Modulation of Vascular Inflammation and Atherosclerosis by Extracellular Vesicles

**DOI:** 10.3389/fcvm.2018.00002

**Published:** 2018-01-22

**Authors:** Emiel P. C. van der Vorst, Renske J. de Jong, Marjo M. P. C. Donners

**Affiliations:** ^1^DZHK (German Centre for Cardiovascular Research), Partner Site Munich Heart Alliance, Munich, Germany; ^2^Institute for Cardiovascular Prevention, Ludwig-Maximilians-University Munich, Munich, Germany; ^3^Center of Allergy Environment (ZAUM), Helmholtz Center, TU Munich, Neuherberg, Germany; ^4^Cardiovascular Research Institute Maastricht (CARIM), Maastricht University, Maastricht, Netherlands

**Keywords:** extracellular vesicles, vascular inflammation, atherosclerosis, proteolytic activity, challenges

## Abstract

Extracellular vesicles (EVs) have emerged as a novel intercellular communication system. By carrying bioactive lipids, miRNAs and proteins they can modulate target cell functions and phenotype. Circulating levels of EVs are increased in inflammatory conditions, e.g., cardiovascular disease patients, and their functional contribution to atherosclerotic disease development is currently heavily studied. This review will describe how EVs can modulate vascular cell functions relevant to vascular inflammation and atherosclerosis, particularly highlighting the role of EV-associated proteolytic activity and effector proteins involved. Furthermore, we will discuss key questions and challenges, especially for EV-based therapeutics.

## Introduction

Extracellular vesicles (EVs) play a crucial physiological and pathophysiological role, as they have been identified as regulators of cell-to-cell communication (1).

Extracellular vesicles are small spherical vesicles, consisting of a lipid bilayer membrane encasing a small organelle-free cytosol, that are released by cells into the extracellular environment (2). It has been shown that most cell types can release EVs, originating from various subcellular membrane compartments (3). Nowadays, EVs are generally classified into three main classes, i.e., exosomes, microvesicles (MVs), and apoptotic bodies (3). Exosomes arise from intracellular compartments called multivesicular bodies (MVBs) and are released by an active process, leading to fusion of these MVBs with the plasma membrane (4). Exosomes typically have a size of 30–100 nm, i.e., representing the smallest subgroup of EVs, and are enriched for tetraspanins (CD9, CD63, and CD81) or other markers, such as flotillin and tumor susceptibility gene 101, which are often used to distinguish them from other populations of EVs (5). The second class of EVs is MVs, which are typically larger in size (ranging from 100 to 1,000 nm) and are produced by budding off directly from the plasma membrane in a process called microvesiculation (5). Microvesiculation involves the externalization of phosphatidylserine (PS) followed by cytoskeleton rearrangement and the formation of membrane curvatures (6, 7). Therefore, MVs membranes are also enriched in PS (detectable by Annexin A5) and the membrane composition resembles that of the parental cell (8). The third type of EVs is apoptotic bodies with a size of >1 μm. These vesicles are released from apoptotic cells through membrane blebbing and therefore contain apoptotic nuclear material (9). However, although the field is rapidly evolving, it is still quite challenging to specifically isolate, characterize, and classify the different populations of vesicles as discussed below.

Extracellular vesicles can cargo a large variety of biomolecules, such as various DNA, RNA, and microRNA species, bioactive lipids, and proteins. The latter include receptor ligands, by which EVs can interact with target cells (2), and proteolytically active enzymes, by which these vesicles can influence many cellular functions (10). This review will give a brief overview on how EVs can modulate vascular cell functions relevant to vascular inflammation and atherosclerosis, particularly highlighting the role of EV-associated proteolytic activity and effector proteins involved. Furthermore, we will discuss key questions and challenges, especially for EV-based therapeutics.

## EVs in Vascular Inflammation and Atherosclerosis

**Figure 1:**
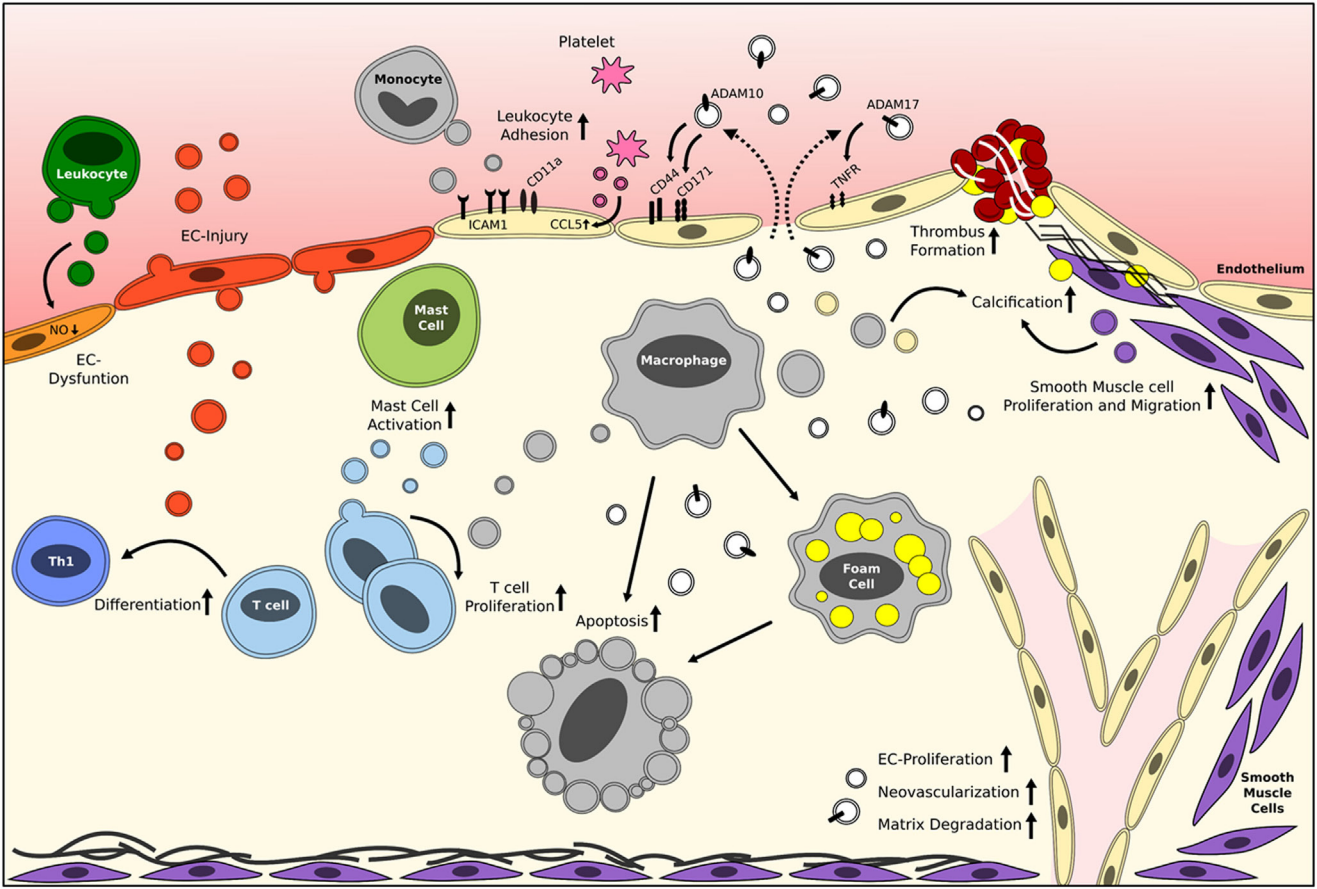
Reported roles of EVs in vascular inflammation and atherosclerosis. Brief schematic representation of the reported effects of circulating cell-derived and plaque-derived EVs on different processes in atherosclerosis development. The mentioned effector molecules are merely examples, and it should be noted that many more exist. White vesicles are of unknown origin/parental cell. EC, endothelial cell; EV, extracellular vesicle. Please refer to Table 1 for more detailed information.

Recent years, great efforts have already been made to elucidate the role of EVs in cardiovascular diseases (CVDs), which is still the major cause of mortality worldwide. CVDs are mainly caused by atherosclerosis, a chronic inflammatory disease initiated by a continuous damage of the vascular endothelium leading to endothelial dysfunction (11). It has already been clearly shown that inflammation and endothelial injury augment the release of EVs (12, 13), generally reflecting the pro-inflammatory state of the parental cell. In addition, EVs influence thrombus formation which can occur after plaque rupture (3). Indeed, atherosclerotic lesions contain and release EVs, derived from leukocytes, platelets, smooth muscle cells (SMCs), and endothelial cells, during all stages of atherosclerosis development (14, 15) (Figure 1). As a consequence, patients with CVD mediated by endothelial damage show significantly elevated levels of circulating cell-derived EVs (16). This observation has therefore also been the starting point to investigate EVs as potential prognostic and diagnostic biomarkers. While most research has focused on the presence and function of MVs, also exosomes have been observed in human atherosclerotic lesions (17), although their functional roles remain largely unexplored.

Several *in vitro* studies clearly show that platelet and leukocyte-derived MVs from unstimulated cells increase the release of pro-inflammatory cytokines from endothelial cells and leukocytes, especially interleukin (IL)-6 and IL-8 (18, 19). Release of these cytokines will inherently promote monocyte adhesion to the endothelium and migration into the atherosclerotic lesions. MVs released from human atherosclerotic plaques were shown to increase the expression of endothelial adhesion molecules such as intercellular adhesion molecule 1 and monocyte adhesion molecule receptors, like CD11a, thereby further augmenting monocyte adhesion (18, 20). Furthermore, CCL5 (RANTES) is transferred from healthy platelet MVs to activated endothelial cells and can thereby enhance leukocyte adhesion (21). Endothelial and leukocyte MVs have also been shown to induce endothelial cell dysfunction, by decreasing the production of nitric oxide (NO) (22, 23). This is the result of an inhibition of the endothelial NO synthase and/or an increase in caveolin-1, increasing local oxidative stress (24, 25). Besides this mediator, MVs can act as potential markers of endothelial dysfunction, as nicely reviewed in Ref. (26). Together, these data clearly show that MVs, derived from various (vascular) cell types, can greatly influence the initiation of atherosclerosis development.

Microvesicles derived from macrophages and fibroblasts have also been implicated in later stages of lesion development, as they can stimulate foam cell formation by lipid/cholesterol uptake in macrophages (27). Furthermore, several reports have indicated that T cell-derived MVs can contribute to monocyte and macrophage apoptosis, *via* two proposed mechanisms (28, 29). The first mechanism involves the phagocytosis of MVs by monocytes and macrophages, leading to an increased cellular content of membrane phospholipids. These phospholipids are likely cleaved by phospholipase A2 into arachidonic acid, which will subsequently result in an increased amount of proapoptotic ceramides inside the cells (28, 29). The second mechanism involves MVs containing caspase-1 or caspase-3, which can induce target cell apoptosis (30, 31). Several studies have shown that MVs also play an important role in lymphocytes, as both human atherosclerotic plaque and *in vitro* generated dendritic cell MVs can stimulate T cell proliferation (32, 33). Most likely this influence is mediated by the presence of major histocompatibility complex class II presence on the MVs secreted from macrophages and dendritic cells (32). Furthermore, endothelial cell-derived MVs can promote lymphocyte differentiation toward a more proatherogenic T helper-1 phenotype as shown by priming of naive T cells with dendritic cells which were matured with endothelial MVs (34). On their turn, activated T cells release MVs that can induce mast cell activation, degranulation, and cytokine release (35). Mast cells are also present in the arterial wall, where they can contribute to atherosclerosis development (36).

Furthermore, MVs have significant effects on plaque stability as they can influence SMC proliferation and migration, *via* protease-activated receptor interaction or various microRNAs (37, 38). In addition, plaque MVs can contribute to matrix degradation as they contain several active proteases (39), which will be discussed in more detail later. This influence on matrix degradation is also one of the mechanisms by which MVs could potentially contribute to intraplaque neovascularization. It has also been shown that human plaque MVs can increase endothelial proliferation, a crucial step in neovascularization, *in vitro* as well as *in vivo* in matrigel plugs (40). During human atherosclerosis development, intimal calcification occurs at different stages of lesion development (41). Moreover, endothelial, SMC, and macrophage-derived EVs are present at the sites of calcification (3), nicely reviewed in Ref. (42). EVs released from SMCs have the potential to stimulate calcification by these same SMCs, mediated by sortilin-dependent regulation of alkaline phosphatase trafficking (43). In addition, EVs enriched in bone morphogenetic protein 2 released from endothelial cells can promote calcification in vascular SMCs (44).

In the latest stages of atherosclerosis, i.e., plaque rupture and thrombosis, MVs can also play an important role. MVs/EVs carry various proteolytic factors that likely contribute to matrix degradation, as shown in cancer (45) and could thereby potentially also influence plaque destabilization. In addition, human plaque MVs have been shown to be particularly prothrombogenic (15). Plaque MVs can contribute to the coagulation pathway *via* two different pathways: the presence of tissue factor on the surface of MVs and the exposure of PS on the outer membrane layer (3, 46). In contrast to MVs, exosomes seem to have antithrombotic effects. Platelet aggregation was suppressed by platelet-derived exosomes by inhibiting platelet CD36 (47). The procoagulant role of MVs is more elaborately reviewed in Ref. (48).

Besides communication between different cells within an atherosclerotic plaque, it is generally assumed that EVs, as they are relatively stable, mediate cross talk with cells at relatively large distances. This is particularly relevant for CVDs, which is widely acknowledged to be a systemic disease, and the basis for the “vulnerable patient concept” (49, 50). Indeed, it has already been long recognized that clinical symptoms in CVD patients (e.g., myocardial infarction or stroke) are often followed by secondary CVD events. Moreover, CVDs are often associated with several comorbidities, e.g., diabetes, chronic kidney disease, non-alcoholic steatohepatitis, small cerebral vessel disease, and heart failure. It is likely, yet it remains to be determined, that EVs play a crucial role in this systemic intercellular communication.

## Proteolytic Enzymes/Effector Molecules in EVs

Extracellular vesicles are known to carry a large amount of bioactive molecules, including proteins/enzymes. Still, relatively little is known on the influence of various (atherogenic) stimuli on EV composition and thus EV function. Proteomic analysis recently identified several proteolytical enzymes in EVs, such as the cell surface-bound sheddases a disintegrin and metalloproteinases (ADAMs), soluble ADAMs with thrombospondin motifs (ADAMTSs), as well as cell surface-bound and soluble matrix metalloproteinases (MMPs) (51).

A disintegrin and metalloproteinases are involved in ectodomain shedding of various transmembrane proteins, thereby regulating cell adhesion, migration, and cell–cell communication (52). ADAM10 and ADAM17 are the best studied members of this family. ADAM17 is considered the primary enzyme for shedding of tumor necrosis factor (TNF), and its receptors (TNFR1 and 2), and the epidermal growth factor receptor ligands (53). On the other hand, ADAM10 is physiologically critical for Notch signaling *via* receptor cleavage (54). ADAMs have been reported to mediate various exosome/MV functions, e.g., by cleavage of EV surface molecules, releasing them as soluble factors in the target cell microenvironment. Indeed, ADAM17 is present on MVs released from atherosclerotic lesions and shown to cleave pro-TNF from these vesicles, which could provide a means to locally release pro-inflammatory mediators at large distances from the cell/site from which the MVs are released (39). In addition, plaque MVs have been shown to increase the shedding of TNF and its receptor (TNFR) from the surface of endothelial cells in an ADAM17-dependent manner (39), further supporting a role for ADAM17^+^ MVs in the regulation of (systemic) vascular inflammation.

Little is known on the role of other EV-associated ADAMs in relation to atherosclerosis. In exosomes, secreted from ovarian carcinoma cells, especially ADAM10 has been shown to be crucially involved in the cleavage of CD171 (L1) and CD44 (55), two important cell adhesion molecules. Cleavage did not only occur in the released exosomes but also already in the earlier phases of vesicle formation in the endosomal compartment. ADAM17 is also able to cleave CD171, although this occurs only at the cell surface demonstrating that different ADAMs are involved in distinct cellular compartments (55), and thus potentially in different EV populations. Other ADAMs such as ADAM15 (56), have also been identified in exosomes. Tumor cell-derived exosomes, enriched in ADAM15, display a high binding affinity for integrin αvβ3 and suppress cell adhesion, migration and growth (56). Exosomes derived from macrophages have also been shown to express ADAM15 and demonstrate described tumor inhibitory effects (56). The functional contribution of ADAM proteases in EVs to CVD disease progression, however, remains to be determined.

ADAMs with thrombospondin motifs are relatively comparable to ADAMs, but have thrombospondin-like motifs instead of transmembrane and cytoplasmic domains and are therefore generally secreted as soluble proteins (45). A large subgroup of ADAMTSs is known as aggrecanases, because they can proteolytically cleave proteoglycans and are involved in cartilage degradation (57). This degradation of cartilage by aggrecanases has been associated with the progression of arthritis (58). Recently, it has been shown that rheumatoid synovial fibroblasts secrete MVs containing aggrecanase activity, most likely mediated by ADAMTS1, ADAMTS4, or ADAMTS5 (59). Synovial fluids in rheumatoid arthritis also contain T cell- and monocyte-derived MVs, which can induce the synthesis of several MMPs in fibroblasts (60). Considering the role of various ADAMTS proteases in inflammation and vascular biology (61, 62), it is likely that EV-associated ADAMTSs are implicated in CVD. However, there are no clear indications for such a role of ADAMTSs in EVs in other pathologies, such as CVDs, yet.

Matrix metalloproteinases are a family of zinc-dependent endopeptidases, which are also crucial to extracellular matrix degradation and cleavage of surface proteins. It has already been shown that EVs released from mouse melanoma cells and human colorectal carcinoma cells have gelatinolytic and collagenolytic activity, indicating the presence of active MMPs (63, 64). Indeed, more recently several MMPs have been detected in EVs derived from tumor cells (45). Interestingly, there is also a positive correlation between the quantity of shed vesicles, the amount of vesicle bound lytic enzymes and the *in vitro* invasive capability of different human cancer cell lines (65). Since MMPs also play a role in CVD (66), a role of MMPs in EVs in CVD can be expected but has surprisingly not been evaluated so far.

## Clinical Potential and Challenges

Targeting EVs seems like a promising novel therapeutic option, where EVs containing RNA, DNA, or proteins involved in disease pathogenesis can be blocked. Blockage of EVs and especially the delivery of their cargo to the target cell can be achieved in various ways, e.g., by inhibiting the vesicle release, uptake or formation [reviewed by El Andaloussi et al. (67)]. Vesicle formation can be suppressed by inhibiting crucial cellular compartments, for instance by ceramide or syndecan proteoglycans blockage. Furthermore, the release of vesicles can be blocked by inhibiting GTPases, which are needed for the fusion of MVBs with the plasma membrane. In addition, EVs could be used as therapeutic delivery tools. For this purpose, both endogenously produced EVs and EVs, which are deliberately packaged with specific components can be used (68). For example, a recent proof of concept study has shown that EVs could deliver specific siRNA to mouse brains (69). In the context of CVD, a recent study has shown that *in vitro* generated endothelial EVs could reduce atherosclerosis formation by the transfer of miRNAs (38). The first clinical trials using EVs have also already been started in the field of antitumor immunotherapy. Two separate phase I trials used Good Manufacturing Practice compatible protocols to isolate EVs from dendritic cells and could show a good feasibility and safety of EV administration in patients (70). The phase II trial that followed unfortunately did not give the expected positive outcomes, but combined these results clearly show the therapeutic potential of EVs.

In addition to their therapeutic use, EVs could also be used as biomarkers as they are also found in several body fluids, such as blood (71) and urine (72), making them easily accessible for prognostic or diagnostic purposes. Emphasizing the prognostic potential, it has already been shown that Cystatin C, Serpin F2, and CD14 MV levels correlate with an increased risk for cardiovascular event and mortality (73). In addition, miRNA content of EVs has already been clearly linked with disease outcome (74). More details about the clinical potential of EVs and their use as biomarkers are recently elaborately reviewed in Ref. (75).

The field of EV research is rapidly progressing, although the EV research complexity and challenges are still considerable (76). EVs represent a very heterogeneous population, both in size and composition. This has led to some confusing and variable nomenclature, although as described before some consensus has already been achieved. Another major difficulty is the presence of non-EV components in preparations of EVs, which have comparable features (77). Currently, various isolation methods are used to isolate EV subtypes, such as differential (ultra)centrifugation, density gradient centrifugation, size exclusion chromatography, and immunocapture. All of these methods result in EV preparations of different composition and especially purity. For example, ultracentrifugation not only pellets EVs but also protein aggregates, while lipoproteins have similar size and density as EVs and are therefore often co-isolated (78). Recently, more confounding factors of ultrafiltration and protein analysis have been identified (79). Since, these different methods have not yet been tested side by side on a single EV sample, reliable quantitative comparisons regarding recovery and purity are difficult. Another interesting point that needs consideration is the influence of medication on EVs. For example, several antiplatelets agents, such as aspirin, can inhibit platelet activation and the related release of MVs (3). Antihypertensive agents have also been shown to reduce circulating platelet- and monocyte-derived MVs (3). In addition, statin therapy influences the composition of endothelial MVs (80). Besides the variety in contaminating factors in the different isolation methods, another major limitation is the unstandardized and often inadequate reporting on the specific methods used. Previously, the International Society for Extracellular Vesicles already introduced the minimal information for studies on EVs guidelines (81). More recently, to further improve the reliability of EV-related data/publications an international consortium developed the EV-TRACK (transparent reporting and centralizing knowledge in EV research) platform (82). This platform urges researchers to report more specific and detailed parameters which are necessary to fully interpret the obtained data and compare different studies. In addition, a recent review gives some methodological guidelines to study EVs (83). All these efforts clearly show the intention to standardize EV procedures, which will also be necessary to advance this research field toward comparable/supportive studies, crucial to pave the way toward clinical trials.

## Concluding Remarks

In the context of CVD and in particular atherosclerosis, a large variety of risk factors and contributing factors have already been identified and are currently targeted to treat this pathology, such as inflammatory molecules and lipids/lipoproteins. Although EVs have already been shown to be of crucial importance in the modulation of vascular inflammation and atherosclerosis (Table 1), at least *in vitro*, little is known on their therapeutic potential for CVD. Moreover, there are still several major limitations that should be overcome, such as detailed characterization and isolation procedures. Therefore, more preclinical studies are necessary before attempting to translate this research field to human medicine. In conclusion, EVs are promising targets for vascular inflammation and atherosclerosis and future research will further elucidate the full potential of such vesicles in disease prognosis, diagnosis and therapy.

**Table 1:** Summarizing described studies supporting the role of EVs in vascular inflammation and atherosclerosis.

Cell origin	Species origin	Study type	Activation stimuli	Main findings	Reference
ECs	Mouse/human	*Ex vivo*/*in vitro*	n.a.	MVs attenuate EC-mediated vasodilation *ex vivo* and reduced NO release *in vitro*	(24)
ECs	Human	*In vitro*/*in vivo*	Hydrogen peroxide	Elevated levels of CD144^+^ EVs reflect EC injury *in vitro* and correlate with CVD risk *in vivo*	(16)
ECs	Human	*In vitro*/*in vivo*	n.a.	MVs correlate with decreased arterial function *in vivo* and decreased NO release *in vitro*	(22)
ECs	Human	*Ex vivo*	n.a.	MVs impaired vasorelaxation and NO production by rat aortic rings	(23)
ECs	Human	*In vivo*/*in vitro*	High glucose	MVs derived from high-glucose ECs impaired endothelial function and increased macrophage infiltration after injection into mice and increased NADPH oxidase activity and ROS levels *in vitro* (compared with MVs from untreated ECs)	(25)
ECs	Human	*In vitro*	Various apoptosis inducer	MVs from apoptotic ECs contain caspase-3	(30)
ECs	Human	*In vitro*	TNF	DCs matured with MVs resulted in priming of naïve T cells toward more proatherogenic T helper-1 phenotype	(34)
ECs	Human	*In vitro*/*in vivo*	KLF2 or shear stress	EVs are enriched in miR-143/145 and control SMC gene expression and phenotype *in vitro* and reduce atherosclerotic lesion formation in mice	(38)
ECs	Human	*In vitro*	TNF	EVs enriched in bone morphogenetic protein 2 promote calcification in SMCs	(46)
SMCs	Human	*In vitro*	n.a.	EVs stimulate calcification of SMCs in a sortilin-dependent manner	(45)
PMNs	Human	*In vitro*	Formyl peptide and phorbol ester	MVs stimulate EC activation and cytokine release	(19)
Monocytes	Human	*In vitro*	Endotoxin	Monocyte-derived MVs contain caspase-1 and induce cell death of SMCs	(31)
Fibroblasts	Mouse	*In vitro*	n.a.	MVs stimulate macrophage foam cell formation, which is enhanced by TLR stimulation	(27)
DCs	Human	*In vitro*	LPS	Released MVs from activated DCs can fuse with resting DCs and activate T cells	(33)
T cells	Human	*In vitro*	Apoptosis inducers	MVs increase macrophage apoptosis and stimulated macrophage MV release	(28)
T cells	Human	*In vitro*	IL-2	MVs perturb lipid homeostasis of macrophages and thereby induce apoptosis	(29)
T cells	Human	*In vitro*	PMA	T cells release MVs that induce mast cell activation, degranulation and cytokine release	(35)
Platelets	Human	*In vitro*	n.a.	MVs increased monocyte adhesion to ECs and chemotaxis	(18)
Platelets	Human	*In vitro*	n.a.	MVs enhance monocyte rolling/arrest by depositing RANTES on ECs	(21)
Platelets	Human	*In vitro*	Thrombin	Exosomes inhibit atherothrombotic processes by reducing CD36-dependent lipid loading of macrophages and by suppressing platelet thrombosis	(49)
Plaques	Human	*Ex vivo*	n.a.	MV are more abundant and thrombogenic in plaques compared with plasma	(15)
Plaques	Human	*Ex vivo*	n.a.	First ultrastructural evidence of plaque exosomes	(17)
Plaques	Human	*Ex vivo*	n.a.	MVs stimulate intercellular adhesion molecule 1-dependent monocyte adhesion	(20)
Plaques	Human	*In vitro*	n.a.	MVs express MHC-I and MHC-II and induce T cell proliferation	(32)
Plaques	Human	*In vitro*	n.a.	ADAM17, present on plaque MVs cleaves pro-TNF from these vesicles Plaque MVs increase TNF shedding and its receptor (TNFR) from ECs	(40)
Plaques	Human	*In vitro*/*in vivo*	n.a.	MVs increased EC proliferation *in vitro* and stimulated *in vivo* angiogenesis in matrigel assays in mice	(41)
Plaques	Human	*Ex vivo*	n.a.	Plaque MVs contribute to the coagulation pathway *via* two different pathways: the presence of tissue factor on the surface of MVs and the exposure of PS	(48)
Plasma	Human	*Ex vivo*	n.a.	Cystatin C, Serpin F2, and CD14 MV levels correlate with an increased risk for cardiovascular event and mortality	(75)
Plasma	Human	*Ex vivo*	n.a.	MVs containing miR-126 and miR-199a predict the occurrence of cardiovascular events	(76)

*DC, dendritic cell; EC, endothelial cell; KLF2, Krüppel-like factor 2; LPS, lipopolysaccharide; MHC, major histocompatibility complex; MV, microvesicle; NO, nitric oxide; PMA, 4-beta-phorbol 12-myristate 13-acetate; PMN, polymorphonuclear leukocytes; PS, phosphatidylserine; SMC, smooth muscle cell; TLR, toll-like receptor; TNF, tumor necrosis factor; EV, extracellular vesicle; IL, interleukin; n.a., not applicable*.

## Author Contributions

EV and RJ: drafting the manuscript. MD: concept and design; drafting the manuscript.

## Conflict of Interest Statement

The authors declare that the research was conducted in the absence of any commercial or financial relationships that could be construed as a potential conflict of interest. The handling editor declared a shared affiliation, although no other collaboration, with one of the authors MD.
